# Blue Food Consumption and Its Relation to Nutrient Intake among Koreans

**DOI:** 10.3390/nu16183128

**Published:** 2024-09-16

**Authors:** Yonghee Suk, Min June Lee, Sunny Ham

**Affiliations:** Department of Food and Nutrition, Yonsei University, 50 Yonsei-ro, Seoul 03722, Republic of Korea; stoney@yonsei.ac.kr (Y.S.); minjlee@yonsei.ac.kr (M.J.L.)

**Keywords:** blue food, seafood, fish, nutrient intake, health benefits

## Abstract

(1) Background: “Blue food” is a recently coined term referring to seafood, emphasizing health benefits and sustainability. This study compares nutrient intake levels between Koreans who consume fish and shellfish and those who do not. (2) Methods: Data from the eighth Korea National Health and Nutrition Examination Survey (2019–2020) were used. A total of 9442 individuals were analyzed (≥1 year old). A complex sample design was applied. (3) Results: Younger individuals aged 9 to 29 consumed less fish and shellfish, while those with higher education and income levels consumed more. Compared with the non-consumption group, individuals in the fish and shellfish consumption group exhibited a higher nutrient density in their diets, excluding total fat, saturated fatty acids, monounsaturated fatty acids, and riboflavin (*p <* 0.01). They also had a higher proportion of nutrient intakes relative to the Recommended Nutrient Intake level for all nutrients than the non-consumption group (*p* < 0.001), particularly in eicosapentaenoic acid and docosahexaenoic acid intakes. Conversely, the non-consumption group had a higher proportion of nutrient intakes below the Estimated Average Requirement compared with the consumption group. (*p* < 0.001). (4) Conclusions: Individuals consuming blue food exhibited higher levels of nutrient intake. Developing strategies to promote the consumption of blue food, considering factors such as age, education, and income, is essential.

## 1. Introduction

“Blue food” deserves recognition for its contributions to human health and environmental protection. The term “blue food” refers to seafood, whether farmed or wild-caught [1]. An editorial in the September 2021 issue of *Nature* suggested promoting the consumption of blue food to help eradicate hunger [2], and since then, blue food has been introduced worldwide. Since its initial introduction by the Korea Marine Institute in 2022 [1], the term “blue food” has garnered official recognition through mentions in press releases from the Korean Ministry of Oceans and Fisheries, elevating its public profile [3,4]. Additionally, global interest in blue food has surged, culminating in the launch of the Aquatic Blue Food Coalition—a collaboration of major fishing nations—during the second UN Ocean Conference on June 2, 2022 [5]. This term has also seen significant scholarly engagement and has been frequently utilized in research [6,7,8]. 

As societies and governments worldwide recognize the importance of seafood consumption for both human health and the environment, they increasingly use the term “blue food” to emphasize these benefits. Consequently, “blue food” has become a global term, used interchangeably with “seafood”. As consumers’ dietary standards rise and public focus on food shifts from subsistence purpose to well-being, public attention is spreading towards sustainable eating habits, human health improvement, and environmental protection values, which blue food meets [9,10,11,12,13]. Remarkably, in terms of gas emissions, fish production for the same weight of protein generates only one-eighth the emissions of red meats [14], underscoring the environmentally friendly profile of blue food. Consequently, global consumption of blue food is on the rise. According to the Food and Agriculture Organization of the United Nations (FAO), global seafood consumption is projected to increase by 20.0% by 2030 compared with 2016, with per capita seafood consumption expected to rise from 20.3 kg in 2016 to 21.5 kg in 2030, an increase of over 1.0 kg [15].

From a nutritional perspective, seafood is a major source of protein and contains many nutrients that are essential for the human body. Numerous studies have confirmed that eicosapentaenoic acid (EPA) and docosahexaenoic acid (DHA), which are abundant in blue foods, offer significant health benefits. The intake of EPA and DHA has been associated with the prevention of cardiovascular diseases [16,17,18] and cognitive impairments [19], lower depression and anxiety rates [20,21], and protection against metabolic disorders throughout life [22]. 

Many countries are formulating policies to enhance public health and sustainability by emphasizing the importance of consuming blue food [23,24,25,26,27]. For instance, Korea’s Ministry of Oceans and Fisheries is actively promoting sustainable fisheries through the introduction of a low-carbon structural transformation in February 2023. This initiative supports the development of processed seafood products and organizes seafood-related events to increase public awareness [23,24,25]. According to the American Dietary Guidelines by the U.S. Department of Agriculture, the “Healthy Eating Pattern” recommends consuming fish two to three times per week [26]. Despite this, 94.0% of children and 80.0% of adults fall short of this recommendation, as noted by the Senior Vice President of Communications and Advocacy at the National Fisheries Institute in 2022 [27]. To further promote blue food consumption, the U.S. Food and Drug Administration has proposed new guidelines for labeling seafood products as “healthy” [27]. 

Blue food is increasingly recognized worldwide as a potential staple for sustainability, driven by growing concerns about the environment and human health. However, this heightened consumer awareness and dietary shift may not be adequately reflected in the performance of the seafood industry [28]. While the food industry overall continues to grow, the seafood market has been relatively shrinking [29] (pp. 2–8). Seafood, the second-largest source of protein in the Korean diet after meat, saw a decrease in consumption from 45.0% in 2013 to 33.0% in 2020, relative to meat consumption [30] (pp. 64–70). The average daily consumption of fish and shellfish per person in Korea also decreased from 47.0 g in 2013 to 40.3 g in 2021, whereas meat consumption increased from 104.4 g to 123.8 g over the same period [31]. The decline in seafood consumption, especially among children and young individuals, is attributed to concerns over safety, high sodium content, and perceptions of seafood as difficult to prepare and having an unpleasant fishy odor [29] (pp. 2–8), [32,33]. A survey of registered dietitians in school food services showed a high incidence of seafood being left uneaten [34] (pp. 56–57).

From 2013 to 2015, Koreans ranked first worldwide in per capita blue food consumption [35]. However, of the total seafood consumed in Korea, 40.5% is seaweed, 35.7% is fish, and 23.7% is shellfish [36], with only fish and shellfish acting as significant protein sources. Recent data show that the most consumed seafood types in 2020 were kelp and brown seaweed [37]. It is crucial to recognize that, apart from seaweed, only fish and shellfish are significant sources of protein and essential nutrients, particularly omega-3 fatty acids. This study focused exclusively on the consumption of fish and shellfish as blue foods. 

Previous studies have examined seafood consumption and preferences [38,39], awareness and preference for seafood [40], the impact of education on preferences and perceptions of seafood [41], and the differing perceptions of raw versus processed seafood in Korea [42]. However, there is a paucity of studies specifically focusing on fish and shellfish. Some preliminary studies include those on the safety of trace or heavy metals contained in fish and shellfish [43,44,45] and diseases linked to the intake of fish and shellfish [46].

The aim of this study was to compare nutrient intake levels between groups that consumed fish and shellfish and those that did not within the Korean population. To this end, three methods were employed. Several methods are widely used to evaluate the nutritional status of the public, such as by comparing nutrient density, which measures the amount of beneficial nutrients per 1000 kcal of food consumed and assesses the nutritional value of a meal. Nutrient density has been frequently used to evaluate meal quality in numerous studies [47,48,49]. Another method involves comparing nutrient intake levels against national dietary guidelines, which include specific metrics such as the Dietary Reference Intakes (DRIs) for Koreans, Estimated Average Requirement (EAR), Recommended Nutrient Intake (RNI), Adequate Intake (AI), and Upper Intake Level (UL). Several studies have applied the RNI and AI to compare nutrient intakes across different age groups [50,51,52]. The third approach uses the EAR to ascertain the prevalence of inadequate nutrient intake, a method widely utilized to assess the nutritional status of groups [53,54,55].

The findings of this study will provide a foundation for developing policies and strategies to reverse the declining consumption of fish and shellfish in Korea. Additionally, the results can serve as evidence to support governmental efforts to promote the consumption of fish and shellfish. 

## 2. Subjects and Methods

### 2.1. Study Design and Population

This study utilized secondary data collected by the Korean government. Specifically, data from the 8th Korea National Health and Nutrition Examination Survey (KNHANES) (2019–2020) were used. This survey was designed to provide representative statistics on the health status, health behaviors, and nutritional status of the Korean population, excluding individuals under the age of 1 year. All participants provided written informed consent, with legal guardians providing consent on behalf of minors. Data analysis employed a stratified cluster sampling method, using city/province, dong/eup/myeon, and housing type (general house/apartment) as stratification variables. The dietary intake of participants was assessed using the 24 h dietary recall method. The total sample size from the national survey was 12,955 individuals.

After excluding ineligible individuals who did not follow a regular diet because of pregnancy (*n* = 40), breastfeeding (*n* = 30), illnesses (*n* = 857), dietary therapy for weight control (*n* = 2270), or other dietary therapy (*n* = 142), those who did not respond or provided “unknown” reasons for their dietary therapy (*n* = 21), and those who showed extreme dietary intakes, either consuming less than 500 kcal *(n* = 87) or more than 5000 kcal (*n* = 63) per day, a total of 9442 individuals remained for analysis. Of these, 7781 were categorized in the fish and shellfish consumption group and 1661 were in the non-consumption group.

The KNHANES survey protocols and procedures were approved by the Institutional Review Board (IRB) of the Korea Disease Control and Prevention Agency (IRB approval numbers: 2018-01-03-C-A, 2018-01-03-2C-A). 

### 2.2. Dietary Intake Assessment

As we obtained the secondary data from KNHANES, the dietary intake assessment was also conducted at the same institution by applying a 24 h recall method. To understand the consumption patterns of fish and shellfish among Koreans, we analyzed the most frequently consumed types of these foods. Additionally, we examined mealtimes, locations, and the presence of companions during fish and shellfish consumption.

To explore the relationship between fish and shellfish intake and nutrient intake levels, the participants were divided into two groups as follows: those who consumed fish and shellfish, and those who did not. Consumption of fish and shellfish was classified according to food group codes, as indicated by the KNHANES. In the KNHANES, food is categorized into 17 groups (cereals, starchy roots, sugars, legumes, nuts and seeds, vegetables, mushrooms, fruits, seaweeds, beverages, alcoholic beverages, condiments, meats, eggs, fish and shellfish, dairy products, and oils and fats). The fish and shellfish category encompasses seafood, excluding seaweed, including both finfish (e.g., cod, mackerel) and shellfish (e.g., abalone, scallops), crustaceans (e.g., shrimp, crab), cephalopods (e.g., squid, octopus), and their byproducts (e.g., roe, internal organs), as well as dried products (e.g., dried pollack). Subsequently, we compared the amounts of nutrient intake between the two groups, such as energy (kcal), protein (g), fat (g), saturated fatty acids (g), monounsaturated fatty acids (g), polyunsaturated fatty acids (g), *n*-3 fatty acids (g), *n*-6 fatty acids (g), EPA (mg), DHA (mg), cholesterol (mg), carbohydrate (mg), calcium (mg), phosphorus (mg), iron (mg), sodium (mg), potassium (mg), vitamin A (μgRAE), thiamine (mg), riboflavin (mg), niacin (mg), folate (μgDEF), and vitamin C (mg).

Several methods were employed to compare nutrient intake between the groups. First, nutrient density was calculated by converting the intake amount of each nutrient per 1000 kcal consumed by the individual. Second, we referred to the DRIs for Koreans [56] and applied age-specific RNI or AI for each nutrient to assess the nutrient intake ratio relative to the RNI or AI for each group. Third, using the DRIs for Koreans [56], we identified the EAR for each nutrient, applied age-specific requirements, and compared the percentage of subjects consuming less than the EAR. All comparisons of nutritional values were made according to age categories and the DRIs for Koreans, and the results were presented together.

### 2.3. Demographic and Socioeconomic Characteristics

The demographic and socioeconomic characteristics of the study participants were calculated based on sex, age, education level (re-classified code), occupation (re-classified code), residence, and monthly household income. Age was reclassified into the following age groups according to the DRIs for Koreans: 1–2, 3–5, 6–8, 9–11, 12–14, 15–18, 19–29, 30–49, 50–64, 65–74, and ≥75 years. Monthly household income was reclassified into the following categories: less than KRW 1,000,000, 1,000,000≤~<2,000,000, 2,000,000≤~<4,000,000, 4,000,000≤~<6,000,000, and ≥8,000,000 million.

### 2.4. Statistical Analysis

SPSS Version 26 (IBM Corp., Armonk, NY, USA) was used to analyze the data. Following the guidelines of the Korea Disease Control and Prevention Agency, a complex sample design analysis was conducted. Descriptive statistics were calculated for all the variables. A generalized linear model was then employed to compare nutrient intake between the fish and shellfish consumption and non-consumption groups. Additionally, to further compare nutrient density and nutrient intake, the data were adjusted and analyzed using age-specific reference nutrient intake values to accommodate variations across different age groups.

## 3. Results

### 3.1. Respondent Profiles

Table 1 presents the demographic and socioeconomic characteristics of the participants. A total of 9442 respondents were analyzed (5237 in 2019 and 4205 in 2020), of whom 51.9% were female and 48.1% were male. The ages of the participants were classified according to the age categories of the DRIs for Koreans. The participants’ residential areas were distributed across the country, with 17.6% residing in Seoul and 25.7% in Gyeonggi Province. 

The fish and shellfish non-consumption group accounted for 18.4%, whereas the consumption group accounted for 81.6% of the total study respondents. There was no difference in the consumption of fish and shellfish between the sexes. The proportion of individuals who did not consume fish and shellfish was higher among those aged 9–29 years, whereas the proportion of individuals who consumed fish and shellfish was higher among those aged 50–74 years. In terms of education, individuals with a college degree or higher had a higher proportion of fish and shellfish consumption, whereas unemployed individuals were less likely to consume these items. Furthermore, individuals with higher income levels tended to have a higher proportion of fish and shellfish consumption.

### 3.2. Consumption Patterns of Fish and Shellfish

**Figure 1:**
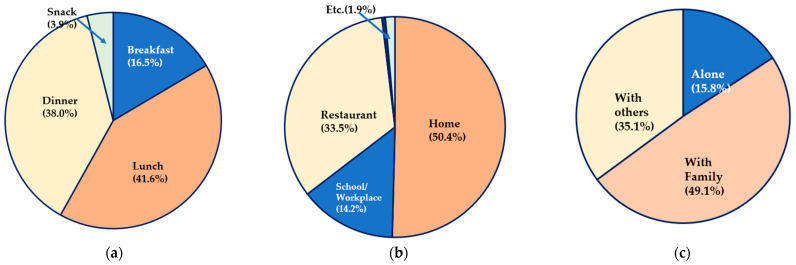
Consumption patterns of fish and shellfish (n = 7781). (**a**) Meal time (weighted %); (**b**) dining place (weighted %); and (**c**) accompanying meals (weighted %).

Figure 1 illustrates the consumption patterns of fish and shellfish. Most respondents reported consuming fish and shellfish during lunch (41.6%) and dinner (38.0%), with home being the most common location for consumption (50.4%). In the consumption group, 33.5% reported consuming fish and shellfish while eating out, with 14.2% consuming them at schools and workplaces. Moreover, 84.2% of the respondents reported consuming fish and shellfish with companions, while only 15.8% reported consuming them alone.

### 3.3. Commonly Consumed (Top 50) Fish and Shellfish among Koreans

Table 2 shows the types of fish and shellfish most frequently consumed by Koreans. Anchovies were the most commonly consumed fish (12.2%), followed by shrimp (8.4%), pickled fish (6.9%), and squid (6.8%). Among shellfish, clams (3.4%) were consumed most frequently, followed by mussels (2.4%) and abalone (1.2%). Processed fish products, such as fish cakes, were also frequently consumed, making up 6.6% of the total.

### 3.4. Comparison of Nutrients between the Fish and Shellfish Consumption and Non-Consumption Groups

Table 3 displays the nutrient density per 1000 kcal for those who consumed fish and shellfish versus those who did not. The nutrient densities of fat, saturated fatty acids, monounsaturated fatty acids, and riboflavin were significantly higher in the non-consumption group (*p* < 0.01), measuring 26.92 g, 9.41 g, 9.02 g, and 0.89 g, respectively. In contrast, these levels in the fish and shellfish consumption group were 23.64 g, 7.73 g, 7.65 g, and 0.86 g, respectively, indicating approximately 1.1–1.2 times higher nutrient density in the consumption group than that in the non-consumption group. Although the nutrient density of *n*-6 fatty acids was not significantly different between the groups, it was higher in the non-consumption group. The densities of other nutrients, including proteins, were significantly higher in the fish and shellfish consumption group, except for polyunsaturated fatty acids and vitamin C (*p* < 0.01). Notably, in the consumption group, the nutrient densities of EPA and DHA were higher by approximately 8.4 and 6.8 times, respectively. The nutrient density of *n*-3 fatty acids, iron, and protein was approximately 1.4 times (0.94 g/0.69 g), 1.2 times (6.05 g/5.26 g), and 1.1 times (36.93 g/34.36 g) higher, respectively, in the consumption group than in the non-consumption group.

### 3.5. Comparison of Nutrient Intake Relative to DRIs between the Fish and Shellfish Consumption and Non-Consumption Groups

Table 4 indicates that the ratios of all nutrients were higher in the fish and shellfish consumption group. Notably, EPA plus DHA intake was 20.38% of the AI in the non-consumption group and 149.97% of the AI in the fish and shellfish consumption group; the consumption group showed approximately 7.4 times higher intakes of EPA plus DHA than the non-consumption group. This represented the most significant difference in nutrient intake between the two groups. Regarding iron, the non-consumption group fell short of the recommended intake (89.08%), whereas the consumption group exceeded it (114.99%).

### 3.6. Comparison of Proportions of Insufficient Nutritional Intakes between the Fish and Shellfish Consumption and Non-Consumption Groups

Table 5 presents the proportion of individuals consuming less than the EAR for each nutrient. For energy intake, those individuals consuming below the estimated energy requirement (EER) were calculated. The proportion of individuals who consumed less than the EAR was higher in the non-consumption group (*p* < 0.001); specifically, the proportions were 42.9% in the non-consumption group and 29.4% in the fish and shellfish consumption group. Regarding iron intake, 52.2% of the non-consumption group and 31.7% of the consumption group fell below the EAR. The lowest proportion of nutrient inadequacy was seen with carbohydrates, showing 3.4% in the non-consumption group and 1.1% in the consumption group. The proportions of inadequate intakes of calcium and vitamin A were high; for calcium, the proportions were 80.1% in the fish and shellfish consumption group and 71.1% in the non-consumption group. For vitamin A, the proportions were 82.5% in the consumption group and 73.3% in the non-consumption group, marking the most significant inadequate intake relative to the EAR in the Korean population.

## 4. Discussion

To our knowledge, this study is the first to compare nutrient intake levels between Korean individuals who consumed fish and shellfish and those who did not. Fish and shellfish are increasingly recognized as “blue food,” valued for both their nutritional benefits and environmental sustainability.

The types of fish and shellfish consumed by participants in the KNHANES were diverse, with the top 50 consumed blue foods distributed across a wide range without focusing on any specific item. According to the 2020 Korean DRIs, major protein sources include pork (second), chicken (third), beef (fourth), eggs (fifth), milk (sixth), and tofu (seventh). Among seafood, anchovies ranked the highest (placed 8th), followed by shrimp (16th), mackerel (17th), squid (18th), and pollack (20th) [56] (p. 144). It is noteworthy how the fish and shellfish categories are split into individual seafood types, whereas land meat categories, such as pork, chicken, and beef, remain the same. Since the results obtained through the single 24 h recall method in the KNHANES are difficult to generalize, tools like Food Frequency Questionnaires (FFQs) are necessary for accurately measuring the consumption of various fish and shellfish. Additionally, a 2022 report by the FAO states that globally consumed seafood includes crab, shrimp, salmon, tuna, and others, which differ from the most commonly consumed seafood in South Korea, indicating that regional preferences play a crucial role in assessing seafood consumption patterns [57].

Individuals who consumed fish and shellfish exhibited significantly higher nutrient densities for almost all nutrients compared with those who did not consume these items (*p* < 0.01). These findings suggest that fish and shellfish are critical in enhancing nutrient intake, likely contributing to the strong interest in seafood among nutrition-conscious individuals both in Korea and across the globe [58,59]. This high nutrient density among fish and shellfish consumers could be a key factor influencing dietary choices.

Higher levels of EPA and DHA were particularly pronounced in the fish and shellfish consumption group. In Korea, pork and beef are the primary sources of fats [30] (pp. 64–70), and as those who did not consume fish and shellfish had higher intakes of most nutrients derived from fats, this group may rely more on meat for protein. However, the high intake of *n*-3 fatty acids, such as EPA and DHA, among fish and shellfish consumers is noteworthy. According to the 2017 KNHANES, the primary sources of EPA and DHA in the Korean diet are blue foods such as mackerel, anchovy, and squid. Additionally, among the top 30 sources of EPA and DHA, most are fish and shellfish, with the remainder being seaweed and processed seafood products [56] (p. 187). Thus, fish and shellfish are critical sources of essential fatty acids. 

While the health benefits of fish and shellfish consumption are well documented, consumption among adolescents and young adults aged 9–29 years is relatively low. Although the middle-aged population (30–74 years old) currently shows high intake levels, the low consumption rates among the younger generation, who will shape future trends, indicate that the future of fish and shellfish consumption may be uncertain. 

Fish and shellfish are primarily consumed at home and more often with company than alone. This pattern aligns with findings from previous studies [60] (pp. 7–22), [61]. Unlike in restaurants or other “eating out” venues, people may choose nutrient-rich foods such as fish and shellfish at home out of concern for the health of household members, leading to home meals being richer in these foods [62]. However, with the rise in single-person households and a preference for convenience, the fish and shellfish industries face potential challenges. 

As food consumption patterns evolve, developing strategies that address these changes becomes essential. People are dining out and ordering delivery more often and prioritize convenience, hygiene, and wellness in their food choices. As food becomes more accessible online, there is an increase in the purchase of processed foods, such as those individually packaged or partially prepared. In response, the blue food sector must adapt to encourage greater consumption of fish and shellfish. Particularly, with the rise in single-person households, there is a growing demand for small-unit packaging and semi-cooked processed fish and shellfish products tailored to individual needs. Blue food must leverage various food processing techniques to enhance its appeal by focusing on convenience in preparation and consumption and by addressing common deterrents such as fishy smells and bones, particularly to appeal to younger consumers. Government agencies and the food industry should actively promote blue food, such as by advertising the benefits of blue food for health and the environment. Furthermore, the food industry should diversify blue food products across consumer purchasing channels [63]. 

It is also important to note that the consumption of seafood is higher among individuals with higher income and education levels. Studies conducted not only in Korea but also in the United States have shown that higher education and income levels are associated with greater seafood consumption [62,64,65]. Individuals with higher income levels may consume more seafood because they perceive it as a more nutritious food [64]. However, seafood is also a relatively expensive source of protein [65], which may pose a financial burden for lower-income groups. Therefore, it is imperative that government policies take these factors into consideration when formulating strategies.

The findings of this study indicate that nutrient intake status was generally better among the fish and shellfish consumption group than the non-consumption group. Because this study focused exclusively on fish and shellfish, we did not compare nutrient intake levels with other food types. However, although other food items could have contributed to the nutrient intake status of the fish and shellfish consumption group, the inclusion or exclusion of a particular food group, such as fish or shellfish, can ultimately affect overall nutrient intake levels. Hence, our findings suggest that regular consumption of fish and shellfish plays a crucial role in promoting a well-balanced and nutritious diet.

Our findings are supported by a U.S. study, which found that adolescents who consumed fish had a significantly higher intake of key nutrients, including proteins, B vitamins, magnesium, selenium, and zinc. However, that study also reported higher calorie, fat, saturated fat, and sodium intake among fish consumers, indicating the need to carefully consider the portion size and preparation methods to avoid potential negative health effects [66]. Food culture varies greatly across countries and can be influenced by many factors, leading to varying results in nutrient intake. Nonetheless, it is evident that consuming fish and shellfish can provide essential nutrients, as shown in the study on U.S. adolescents. 

Although no previous studies have compared nutrient intake between individuals who consume fish and shellfish and those who do not, research has been conducted for other foods conventionally labeled as “healthy,” such as fruits, beans, and nuts. Individuals who consumed fruit exhibited higher dietary and meal quality than those who did not [67]. Those with sufficient fruit intake also showed higher recommended intake ratios of dietary fiber, vitamin D, vitamin C, riboflavin, vitamin B_6_, folic acid, calcium, potassium, and magnesium [68]. Another study on college students that compared nutrient intake levels based on black bean consumption found that black bean consumers had higher intake levels of all nutrients, including calories [69]. A U.S. study found that nut consumption is associated with improved nutrient intake and diet quality [70]. These findings suggest that individuals who choose “healthy” foods tend to have better overall nutrient intakes. As a result, people who eat healthy foods also frequently consume high-quality foods, which may be termed a “coupling of healthy and high-quality food consumption.” Further research on various food groups is necessary to better understand the nutritional status of food intake in South Korea.

Notably, excessive consumption of fish and shellfish may also be harmful because of the presence of heavy metals and environmental pollutants. Frequent seafood consumption can lead to higher levels of mercury in the bloodstream [71]. As heavy metals are difficult to excrete once they enter the body, regulatory agencies such as the FDA in the U.S. and the Korean Ministry of Food and Drug Safety have established guidelines for pregnant women and infants regarding the consumption of deep-sea fish with high levels of heavy metals [72,73]. Additionally, because of heightened public concern following the recent discharge of contaminated water from Fukushima, thorough monitoring is essential. Despite these concerns, many studies have shown that the benefits of consuming fish generally outweigh the risks, suggesting that blue food could be considered sustainable for the future [74,75]. Thus, promoting the consumption of fish and shellfish is necessary, but it must be accompanied by measures that reassure the public.

This study utilized the 2019–2020 KNHANES dataset to explore the nutritional benefits of fish and shellfish. However, this dataset has certain limitations. The KNHANES food intake survey employed the 24 h dietary recall method. Owing to the restricted timeframe of the data, it could not be assumed that individuals who did not consume fish and shellfish on the survey day typically refrained from consuming these foods on other days. Our results clearly indicated that individuals that consumed fish and shellfish on the survey day had an overall better nutrient intake than those who did not. However, to draw more robust conclusions regarding the relationship between fish and shellfish consumption and nutrient intake, future studies should conduct seasonal food intake surveys or use an FFQ that accurately reflects average food intake. 

## 5. Conclusions

This study demonstrates that Korean individuals who consumed blue food had higher nutrient intake levels for most nutrients than those who did not. Given that blue food consumption varies by age, education level, and income, it is vital to consider these factors from multiple angles when developing products and implementing policies to promote seafood intake. The findings suggest that promoting fish and shellfish intake, especially among younger populations and those with lower consumption levels, could play a crucial role in improving overall public health. Policymakers and health educators should consider strategies such as targeted awareness campaigns and incorporating blue food-friendly dietary guidelines to encourage healthier eating habits.

## Figures and Tables

**Table 1:** Demographic and socioeconomic characteristics of the study participants based on fish and shellfish consumption.

Variable	Category	n (Weighted %)			
		Non-Consumption Group	Consumption Group	Total	*χ*^2^*/F* Value
Sex	Male	769 (18.3)	3666 (81.7)	4435 (48.1)	0.24/0.17
	Female	892 (18.5)	4115 (81.5)	5007 (51.9)	
	Sum	1661 (18.4)	7781 (81.6)	9442 (100.0)	
Age (years)	1~2	42 (19.3)	159 (80.7)	201 (1.7)	0.24/0.17 ***
	3~5	58 (16.0)	325 (84.0)	383 (3.0)	
	6~8	74 (16.5)	367 (83.5)	441 (3.6)	
	9~11	77 (21.0)	322 (79.0)	399 (3.3)	
	12~14	86 (26.1)	241 (73.9)	327 (3.1)	
	15~18	99 (25.3)	259 (74.7)	358 (4.6)	
	19~29	258 (28.1)	634 (71.9)	892 (13.9)	
	30~49	351 (16.8)	1872 (83.2)	2223 (28.2)	
	50~64	283 (13.7)	1744 (86.3)	2027 (22.7)	
	65~74	156 (13.3)	1076 (86.7)	1232 (8.7)	
	Over 75	177 (19.5)	782 (80.5)	959 (7.1)	
	Sum	1661 (18.4)	7781 (81.6)	9442 (100.0)	
Education level	≦Elementary school	579 (20.2)	2437 (79.8)	3016 (27.2)	35.84/7.13 ***
	Middle school	157 (19.5)	719 (80.5)	876 (9.7)	
	High school	390 (19.4)	1783 (80.6)	2173 (29.9)	
	≧College graduate	338 (14.5)	2019 (85.5)	2357 (33.2)	
	Sum	1464 (18.0)	6958 (82.0)	8422 (100.0)	
Area	Seoul	304 (18.8)	1,372 (81.2)	1676 (17.6)	51.82/1.58
	Busan	90 (14.7)	530 (85.3)	620 (6.6)	
	Daegu	53 (17.3)	339 (82.7)	392 (4.7)	
	Incheon	78 (19.0)	364 (81.0)	442 (5.8)	
	Gwangju	57 (20.0)	244 (80.0)	301 (3.2)	
	Daejeon	51 (17.4)	228 (82.6)	279 (3.2)	
	Ulsan	32 (17.6)	173 (82.4)	205 (2.2)	
	Sejong	23 (16.2)	155 (83.8)	178 (0.7)	
	Gyeonggi-do	468 (20.7)	1916 (79.3)	2384 (25.7)	
	Gangwon-do	61 (19.3)	272 (80.7)	333(3.1)	
	Chungcheongbuk-do	69 (22.3)	228 (77.7)	297(3.1)	
	Chungcheongnam-do	65 (18.4)	282 (81.6)	347 (4.2)	
	Jeollabuk-do	53 (12.2)	265 (87.8)	318 (3.4)	
	Jeollanam-do	49 (13.6)	288 (86.4)	337 (3.6)	
	Gyeongsangbuk-do	100 (22.4)	416 (77.6)	516 (5.0)	
	Gyeongnam-do	74 (12.6)	553 (87.4)	627 (6.7)	
	Jejudo	34 (20.2)	156 (79.8)	190 (1.2)	
	Sum	1661 (18.4)	7781 (81.6)	9442 (100.0)	
Occupation	Administrator, professional	135 (15.8)	720 (84.2)	855 (14.7)	50.29/5.70 ***
	Office work	83 (12.9)	554 (87.1)	637(10.7)	
	Service/sale	150 (18.0)	680 (82.0)	830(13.0)	
	Agricultural and fisheries	33 (11.9)	207 (88.1)	240(2.4)	
	Technician, device/machine operator	85 (13.5)	603 (86.5)	688(11.9)	
	Simple labor	96 (16.6)	544 (83.4)	640(7.9)	
	Inoccupation (housewife, student)	558 (21.2)	2296 (78.8)	2854(39.4)	
	Sum	1140 (17.6)	5604 (82.4)	6744(100.0)	
Household monthly income (KWR)	1,000,000≥	239 (24.9)	810 (75.1)	1049 (8.4)	31.16/3.17 **
	1,000,000≤~<2,000,000	185 (18.9)	909 (81.1)	1094 (9.8)	
	2,000,000≤~<4,000,000	419 (18.3)	1996 (81.7)	2415 (25.2)	
	4,000,000≤~<6,000,000	397 (18.4)	1974 (81.6)	2271 (25.8)	
	6,000,000≤~<8,000,000	189 (17.0)	1032 (83.0)	1221 (14.0)	
	8,000,000≤	220 (15.8)	1129 (84.2)	1349 (16.7)	
	Sum	1,649 (18.4)	7750 (81.6)	9399 (100.0)	

Cross tabulation analysis; ** *p <* 0.01, *** *p <* 0.001.

**Table 2:** Commonly consumed (top 50 highest) fish and shellfish among Koreans.

Ranking	Name of Fish and Shellfish	n (Weighted%)	Ranking	Name of Fish and Shellfish	n (Weighted%)
1	Anchovy	4447 (12.2)	26	Warty sea squirt	220 (0.7)
2	Fish broth	4331 (13.1)	27	Conch	184 (0.6)
3	Shrimp	2643 (8.4)	28	Halibut	148 (0.4)
4	Pickled fish	2376 (6.9)	29	Webfoot octopus	143 (0.4)
5	Fish sauce (liquid)	2196 (6.2)	30	Cod	142 (0.5)
6	Squid	2117 (6.8)	31	Filefish	140 (0.4)
7	Fish cake	2005 (6.6)	32	Mudfish	132 (0.4)
8	Clam	1062 (3.4)	33	Flatfish	129 (0.4)
9	Mackerel	973 (2.9)	34	Freshwater snail	123 (0.4)
10	Pollock	898 (2.5)	35	Skate	118 (0.3)
11	Mussel	762 (2.4)	36	Salmon	114 (0.4)
12	Horse mackerel	694 (2.3)	37	Fish cake broth	113 (0.4)
13	Crab	674 (2.0)	38	Pickled fish roe	107 (0.3)
14	Fish cake (crab flavor)	564 (1.7)	39	Seabream	94 (0.2)
15	Yellow croaker	508 (1.3)	40	Scallop	91 (0.3)
16	Fish roe	460 (1.4)	41	Cockle	84 (0.2)
17	Hairtail	434 (1.1)	42	White bait	84 (0.2)
18	Abalone	399 (1.2)	43	Rock fish	84 (0.3)
19	Crab stick	390 (1.5)	44	Smooth clam	82 (0.3)
20	Eel	361 (1.1)	45	Sea cucumber	81 (0.3)
21	Octopus	327 (1.1)	46	Japanese Spanish mackerel	72 (0.2)
22	Tuna	288 (0.9)	47	Anglerfish	70 (0.2)
23	Oyster	285 (0.8)	48	Fish sausage	66 (0.2)
24	Small octopus	269 (0.9)	49	Blow fish	64 (0.2)
25	Pacific saury	225 (0.7)	50	Fishery byproduct	62 (0.2)

**Table 3:** Comparison of nutrient density (per 1000 kcal of energy intake) between the fish and shellfish consumption and non-consumption groups.

	Non-Consumption (M ± S.E.)	Consumption (M ± S.E.)	Overall Average (M ± S.E.)	Wald F	*t*-Value
Protein (g)	34.36 ± 0.33	36.93 ± 0.15	36.46 ± 0.15	56.15	241.56 ***
Fat (g)	26.92 ± 0.40	23.64 ± 0.18	24.25 ± 0.17	62.69	133.77 ***
Saturated fatty acids (g)	9.41 ± 0.15	7.73 ± 0.07	8.04 ± 0.07	108.62	110.71 ***
Monounsaturated fatty acids (g)	9.02 ± 0.16	7.65 ± 0.07	7.91 ± 0.07	72.41	107.43 ***
Polyunsaturated fatty acids (g)	5.93 ± 0.12	5.97 ± 0.05	5.97 ± 0.05	0.12	131.10
*n*-3 fatty acids (g)	0.69 ± 0.02	0.94 ± 0.01	0.90 ± 0.01	138.73	76.04 ***
*n*-6 fatty acids (g)	5.22 ± 0.11	5.02 ± 0.04	5.06 ± 0.04	3.23	125.04
EPA (mg) ^1^	7.70 ± 0.63	64.84 ± 2.04	54.33 ± 1.70	681.26	31.86 ***
DHA (mg) ^2^	20.17 ± 1.19	137.54 ± 4.26	115.94 ± 3.57	685.54	32.30 ***
Cholesterol (mg)	123.13 ± 3.57	137.13 ± 1.41	134.55 ± 1.31	13.00	97.33 ***
Carbohydrate (g)	147.61 ± 1.11	151.19 ± 0.54	150.53 ± 0.51	9.59	282.50 **
Calcium (mg)	252.06 ± 4.24	274.83 ± 2.12	270.64 ± 2.00	25.54	129.61 ***
Phosphorus (mg)	498.30 ± 3.92	551.97 ± 1.91	542.10 ± 1.85	171.61	288.61 ***
Iron (mg)	5.26 ± 0.10	6.05 ± 0.04	5.90 ± 0.04	50.83	142.15 ***
Sodium (mg)	1570.09 ± 23.57	1771.10 ± 12.18	1734.12 ± 10.97	58.10	145.36 ***
Potassium (mg)	1312.03 ± 15.67	1445.45 ± 7.56	1420.90 ± 7.23	66.88	191.10 ***
Vitamin A (μgRE) ^3^	179.51 ± 5.12	202.00 ± 2.89	197.86 ± 2.74	18.42	69.80 ***
Thiamine (mg)	0.66 ± 0.01	0.69 ± 0.00	0.68 ± 0.00	8.28	142.52 **
Riboflavin (mg)	0.89 ± 0.01	0.86 ± 0.01	0.86 ± 0.01	6.91	165.40 **
Niacin (mg)	6.02 ± 0.09	6.57 ± 0.04	6.47 ± 0.04	36.75	180.85 ***
Folate (μgDFE) ^4^	148.24 ± 2.37	166.09 ± 1.32	162.80 ± 1.25	52.25	126.16 ***
Vitamin C (mg)	32.55 ± 1.52	35.74 ± 0.81	35.16 ± 0.73	3.57	44.39

t: Independent samples *t*-test (generalized linear model); ** *p <* 0.01, *** *p <* 0.001. ^1^ EPA: eicosapentaenoic acid, ^2^ DHA: docosahexaenoic acid, ^3^ μgRE: micrograms of Retinol Equivalents, ^4^ μgDFE: micrograms of Dietary Folate Equivalents.

**Table 4:** Nutrient intake compared to DRIs (Dietary Reference Intakes) between the fish and shellfish consumption and non-consumption groups.

	Non-Consumption (% ± S.E)	Consumption (% ± S.E)	Overall Average (% ± S.E)	Wald F	*t*-Value
Energy	86.08 ± 1.06	95.70 ± 0.57	93.93 ± 0.53	71.33	167.72 ***
Protein	113.04 ± 1.89	133.38 ± 1.15	129.64 ± 1.07	98.34	116.24 ***
EPA ^1^ + DHA ^2^	20.38 ± 1.35	149.97 ± 4.77	126.05 ± 3.99	677.32	31.48 ***
Carbohydrate	193.36 ± 2.73	216.21 ± 1.30	212.00 ± 1.24	62.87	165.83 ***
Calcium	55.86 ± 1.22	67.22 ± 0.61	65.36 ± 0.57	73.09	109.74 ***
Phosphorus	118.06 ± 1.91	146.33 ± 1.16	141.13 ± 1.04	165.13	126.45 ***
Iron	89.08 ± 2.14	114.99 ± 1.15	110.22 ± 1.08	121.46	99.74 ***
Sodium	191.68 ± 3.67	237.62 ± 2.09	229.16 ± 1.91	124.44	113.63 ***
Potassium	65.58 ± 1.05	79.19 ± 0.56	76.69 ± 0.52	138.15	141.39 ***
Vitamin A	47.30 ± 1.46	59.12 ± 0.93	56.94 ± 0.84	53.85	63.88 ***
Thiamine	109.12 ± 2.13	124.95 ± 1.14	122.04 ± 1.05	45.65	109.35 ***
Riboflavin	123.82 ± 2.40	131.77 ± 1.15	130.31 ± 1.06	9.32	114.85 **
Niacin	75.84 ± 1.51	90.47 ± 0.82	87.77 ± 0.76	80.05	110.96 ***
Folate	66.62 ± 1.17	82.32 ± 0.67	79.43 ± 0.63	147.30	121.98 ***
Vitamin C	59.93 ± 2.86	72.20 ± 1.69	69.94 ± 1.53	14.70	42.64 ***

DRIs (Dietary Reference Intakes): Energy is EER (estimated energy requirement), EPA+DHA, sodium, and potassium are Adequate Intake (AI), Recommended Nutrition Intake (RNI) of other nutrients. t: Independent samples t-test (generalized linear model); ** *p <* 0.01, *** *p <* 0.001. The average values were presented after comparing DRIs (Dietary Reference Intakes) by sex and age. ^1^ EPA: eicosapentaenoic acid, ^2^ DHA: docosahexaenoic acid.

**Table 5:** Comparison of proportions with insufficient nutrient intake between the fish and shellfish consumption and non-consumption groups.

	Non Consumption n (Weighted%)	Consumption n (Weighted%)	Overall Average n (Weighted%)	*χ* ^2^
Energy	671 (42.9)	2278 (29.4)	2949 (31.9)	118.88 ***
Protein	573 (34.1)	1659 (19.6)	2232 (22.3)	172.24 ***
Carbohydrate	59 (3.4)	84 (1.1)	143 (1.5)	151.46 ***
Calcium	1328 (80.1)	5558 (71.1)	6886 (72.7)	58.00 ***
Phosphorus	554 (33.4)	1356 (16.4)	1910 (19.5)	260.26 ***
Iron	845 (52.2)	2431 (31.7)	3276 (35.5)	259.07 ***
Vitamin A	1357 (82.5)	5681 (73.7)	7378 (75.3)	58.40 ***
Thiamine	684 (41.7)	2134 (27.7)	2818 (30.2)	133.20 ***
Riboflavin	568 (34.2)	2168 (27.3)	2736 (28.6)	32.36 ***
Niacin	999 (60.9)	3638 (45.5)	4637 (48.4)	133.64 ***
Folate	915 (61.5)	3196 (46.0)	4111 (48.9)	135.22 ***
Vitamin C	1057 (69.8)	4147 (60.1)	5204 (61.9)	56.53 ***

Insufficient nutrient intake: For energy, the proportion of individuals whose intake was below 75% of the estimated energy requirement (EER), and for other nutrients, the proportion of individuals whose intake was below the Estimated Average Requirement (EAR). *** *p <* 0.001 The average values are presented after comparing nutrient intake standards by sex and age.

## Data Availability

The datasets analyzed during the current study are available in the Korea Centers for Disease Control and Prevention database, which can be accessed through the following link: [Korea National Health and Nutrition Examination Survey] (https://knhanes.kdca.go.kr/knhanes/sub03/sub03_02_05.do accessed on 5 July 2022). Registration for membership is required to utilize the raw data, and this right extends to researchers.
